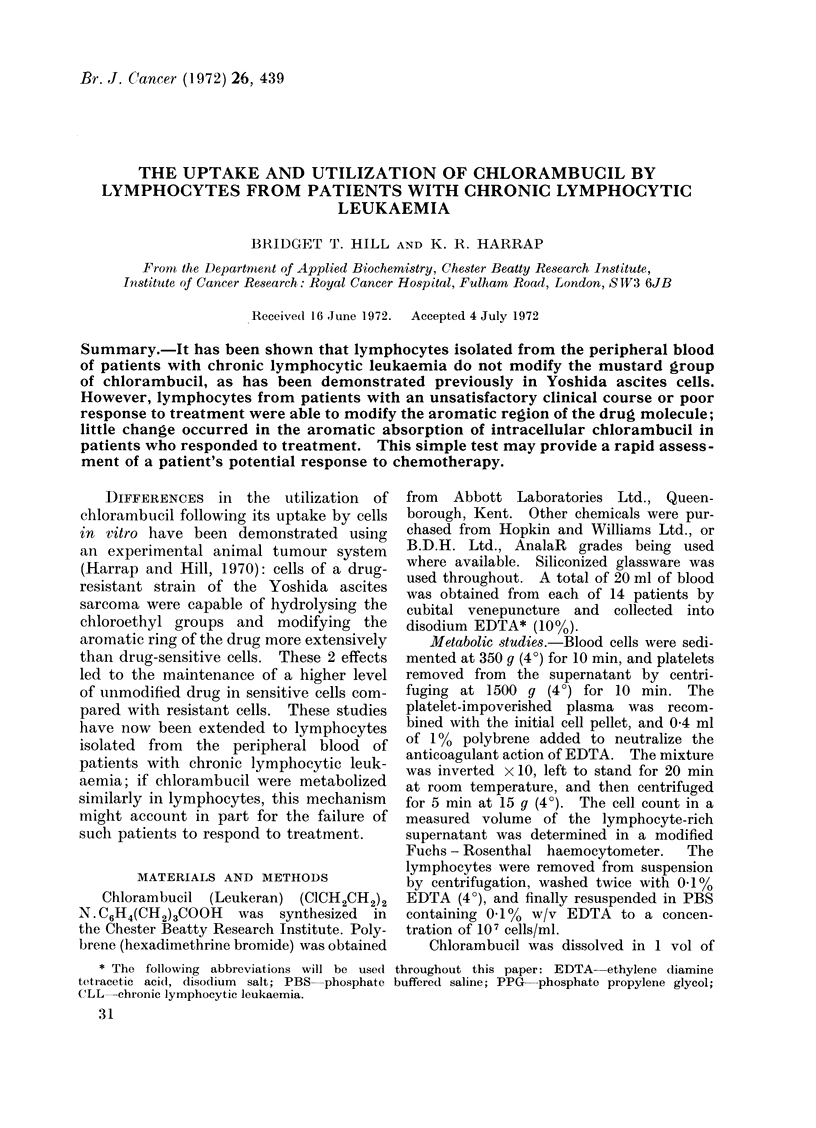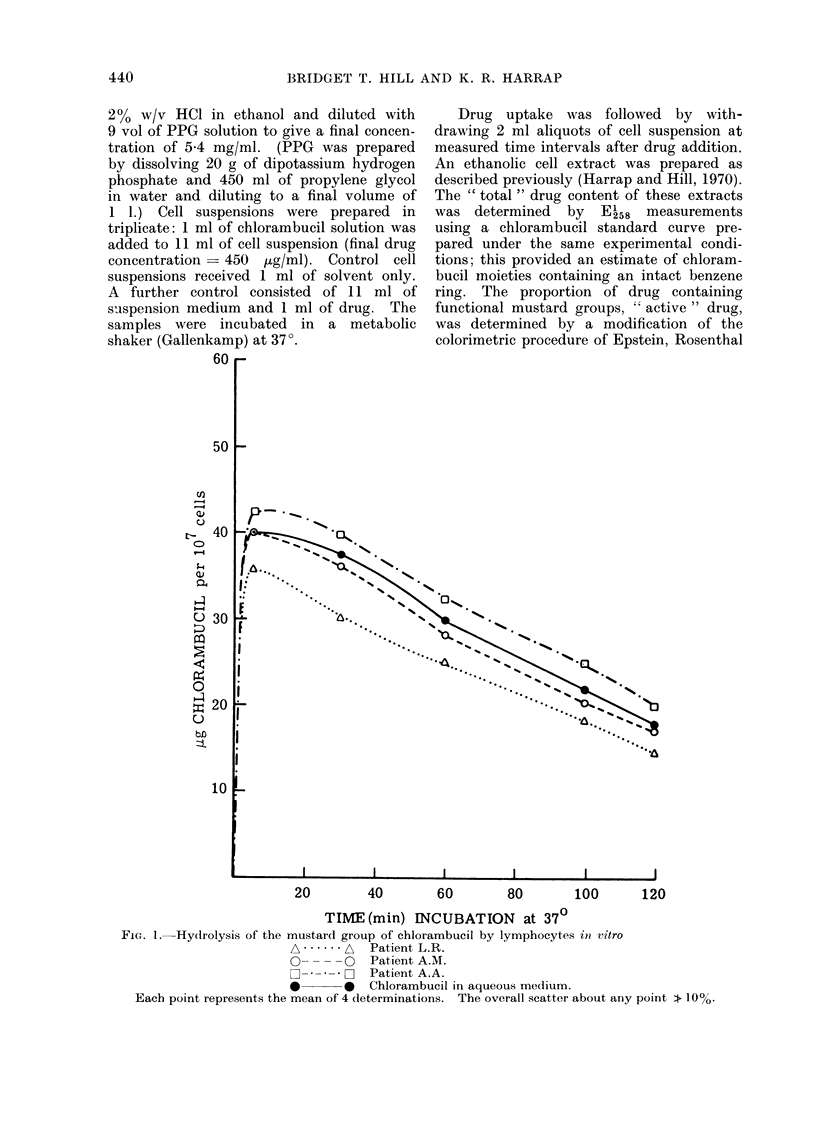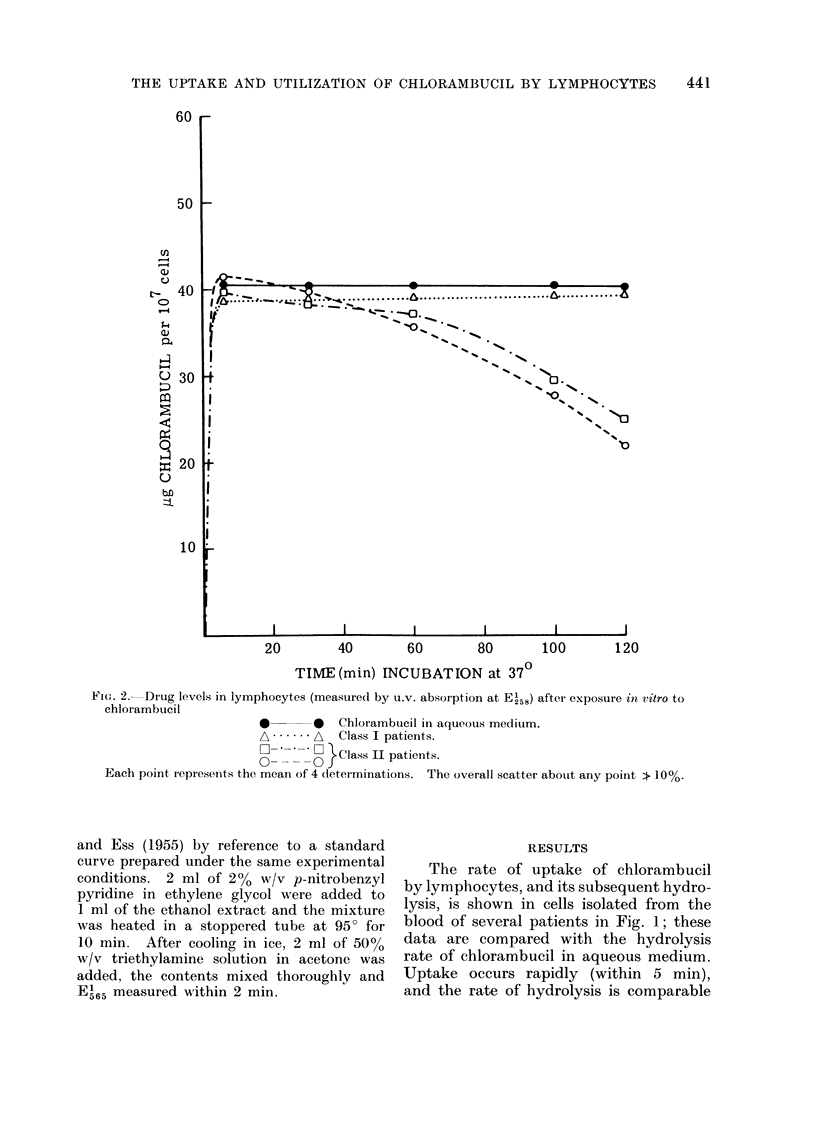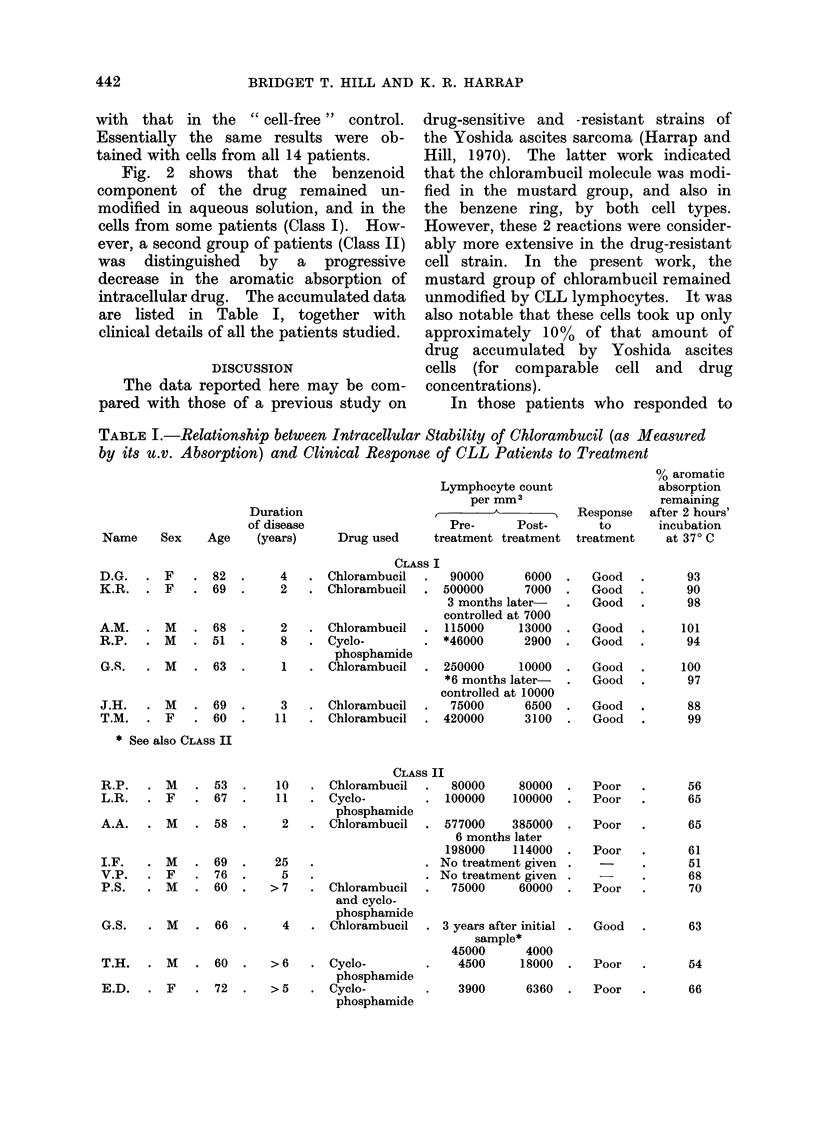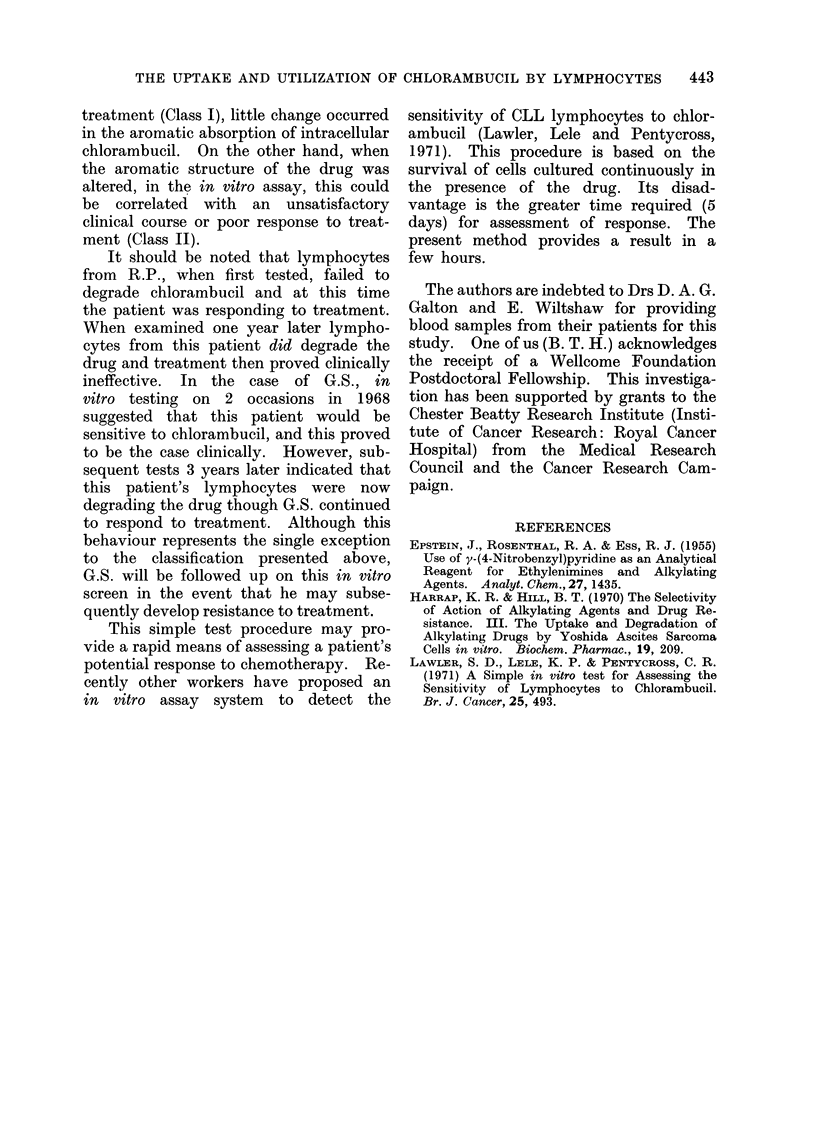# The Uptake and Utilization of Chlorambucil by Lymphocytes from Patients with Chronic Lymphocytic Leukaemia

**DOI:** 10.1038/bjc.1972.60

**Published:** 1972-12

**Authors:** Bridget T. Hill, K. R. Harrap

## Abstract

It has been shown that lymphocytes isolated from the peripheral blood of patients with chronic lymphocytic leukaemia do not modify the mustard group of chlorambucil, as has been demonstrated previously in Yoshida ascites cells. However, lymphocytes from patients with an unsatisfactory clinical course or poor response to treatment were able to modify the aromatic region of the drug molecule; little change occurred in the aromatic absorption of intracellular chlorambucil in patients who responded to treatment. This simple test may provide a rapid assessment of a patient's potential response to chemotherapy.


*Br. J. Cancer* (1972) **26**, 439

### THE UPTAKE AND UTILIZATION OF CHLORAMBUCIL BY LYMPHOCYTES FROM PATIENTS WITH CHRONIC LYMPHOCYTIC LEUKAEMIA

BRIDGET T. HILL AND K. R. HARRAP

*From the Department of Applied Biochemistry, Chester Beatty Research Institute, Institute of Cancer Research: Royal Cancer Hospital, Fulham Road, London, SW3 6JB*

Received 16 June 1972. Accepted 4 July 1972

**Summary.—It has been shown that lymphocytes isolated from the peripheral blood of patients with chronic lymphocytic leukaemia do not modify the mustard group of chlorambucil, as has been demonstrated previously in Yoshida ascites cells. However, lymphocytes from patients with an unsatisfactory clinical course or poor response to treatment were able to modify the aromatic region of the drug molecule; little change occurred in the aromatic absorption of intracellular chlorambucil in patients who responded to treatment. This simple test may provide a rapid assessment of a patient's potential response to chemotherapy.**

DIFFERENCES in the utilization of chlorambucil following its uptake by cells *in vitro* have been demonstrated using an experimental animal tumour system (Harrap and Hill, 1970): cells of a drug-resistant strain of the Yoshida ascites sarcoma were capable of hydrolysing the chloroethyl groups and modifying the aromatic ring of the drug more extensively than drug-sensitive cells. These 2 effects led to the maintenance of a higher level of unmodified drug in sensitive cells compared with resistant cells. These studies have now been extended to lymphocytes isolated from the peripheral blood of patients with chronic lymphocytic leukaemia; if chlorambucil were metabolized similarly in lymphocytes, this mechanism might account in part for the failure of such patients to respond to treatment.

### MATERIALS AND METHODS

Chlorambucil (Leukeran) $(ClCH_2CH_2)_2N.C_6H_4(CH_2)_3COOH$ was synthesized in the Chester Beatty Research Institute. Polybrene (hexadimethrine bromide) was obtained from Abbott Laboratories Ltd., Queenborough, Kent. Other chemicals were purchased from Hopkin and Williams Ltd., or B.D.H. Ltd., AnalaR grades being used where available. Siliconized glassware was used throughout. A total of 20 ml of blood was obtained from each of 14 patients by cubital venepuncture and collected into disodium EDTA* (10%).

*Metabolic studies.*—Blood cells were sedimented at 350 *g* (4°) for 10 min, and platelets removed from the supernatant by centrifuging at 1500 *g* (4°) for 10 min. The platelet-impoverished plasma was recombined with the initial cell pellet, and 0·4 ml of 1% polybrene added to neutralize the anticoagulant action of EDTA. The mixture was inverted ×10, left to stand for 20 min at room temperature, and then centrifuged for 5 min at 15 *g* (4°). The cell count in a measured volume of the lymphocyte-rich supernatant was determined in a modified Fuchs - Rosenthal haemocytometer. The lymphocytes were removed from suspension by centrifugation, washed twice with 0·1% EDTA (4°), and finally resuspended in PBS containing 0·1% w/v EDTA to a concentration of $10^7$ cells/ml.

Chlorambucil was dissolved in 1 vol of

* The following abbreviations will be used throughout this paper: EDTA—ethylene diamine tetracetic acid, disodium salt; PBS—phosphate buffered saline; PPG—phosphate propylene glycol; CLL—chronic lymphocytic leukaemia.

2% w/v HCl in ethanol and diluted with 9 vol of PPG solution to give a final concentration of 5·4 mg/ml. (PPG was prepared by dissolving 20 g of dipotassium hydrogen phosphate and 450 ml of propylene glycol in water and diluting to a final volume of 1 l.) Cell suspensions were prepared in triplicate: 1 ml of chlorambucil solution was added to 11 ml of cell suspension (final drug concentration = 450 μg/ml). Control cell suspensions received 1 ml of solvent only. A further control consisted of 11 ml of suspension medium and 1 ml of drug. The samples were incubated in a metabolic shaker (Gallenkamp) at 37°.

Drug uptake was followed by withdrawing 2 ml aliquots of cell suspension at measured time intervals after drug addition. An ethanolic cell extract was prepared as described previously (Harrap and Hill, 1970). The " total " drug content of these extracts was determined by $E^{1}_{258}$ measurements using a chlorambucil standard curve prepared under the same experimental conditions; this provided an estimate of chlorambucil moieties containing an intact benzene ring. The proportion of drug containing functional mustard groups, " active " drug, was determined by a modification of the colorimetric procedure of Epstein, Rosenthal

FIG. 1.—Hydrolysis of the mustard group of chlorambucil by lymphocytes *in vitro*

△······△ Patient L.R.

○– – – –○ Patient A.M.

□–·–·–·□ Patient A.A.

●———● Chlorambucil in aqueous medium.

Each point represents the mean of 4 determinations. The overall scatter about any point ≯ 10%.

FIG. 2.—Drug levels in lymphocytes (measured by u.v. absorption at $E^1_{258}$) after exposure *in vitro* to chlorambucil

●———● Chlorambucil in aqueous medium.
△······△ Class I patients.
□-·-·-·□ } Class II patients.
○- - - -○ }

Each point represents the mean of 4 determinations. The overall scatter about any point ≯ 10%.

and Ess (1955) by reference to a standard curve prepared under the same experimental conditions. 2 ml of 2% w/v *p*-nitrobenzyl pyridine in ethylene glycol were added to 1 ml of the ethanol extract and the mixture was heated in a stoppered tube at 95° for 10 min. After cooling in ice, 2 ml of 50% w/v triethylamine solution in acetone was added, the contents mixed thoroughly and $E^1_{565}$ measured within 2 min.

RESULTS

The rate of uptake of chlorambucil by lymphocytes, and its subsequent hydrolysis, is shown in cells isolated from the blood of several patients in Fig. 1; these data are compared with the hydrolysis rate of chlorambucil in aqueous medium. Uptake occurs rapidly (within 5 min), and the rate of hydrolysis is comparable with that in the "cell-free" control. Essentially the same results were obtained with cells from all 14 patients.

Fig. 2 shows that the benzenoid component of the drug remained unmodified in aqueous solution, and in the cells from some patients (Class I). However, a second group of patients (Class II) was distinguished by a progressive decrease in the aromatic absorption of intracellular drug. The accumulated data are listed in Table I, together with clinical details of all the patients studied.

### DISCUSSION

The data reported here may be compared with those of a previous study on drug-sensitive and -resistant strains of the Yoshida ascites sarcoma (Harrap and Hill, 1970). The latter work indicated that the chlorambucil molecule was modified in the mustard group, and also in the benzene ring, by both cell types. However, these 2 reactions were considerably more extensive in the drug-resistant cell strain. In the present work, the mustard group of chlorambucil remained unmodified by CLL lymphocytes. It was also notable that these cells took up only approximately 10% of that amount of drug accumulated by Yoshida ascites cells (for comparable cell and drug concentrations).

In those patients who responded to

TABLE I.—*Relationship between Intracellular Stability of Chlorambucil (as Measured by its u.v. Absorption) and Clinical Response of CLL Patients to Treatment*

| Name | Sex | Age | Duration of disease (years) | Drug used | Lymphocyte count per mm³: Pre-treatment | Post-treatment | Response to treatment | % aromatic absorption remaining after 2 hours' incubation at 37° C |
|---|---|---|---|---|---|---|---|---|
| | | | | CLASS I | | | | |
| D.G. | F | 82 | 4 | Chlorambucil | 90000 | 6000 | Good | 93 |
| K.R. | F | 69 | 2 | Chlorambucil | 500000 | 7000 | Good | 90 |
| | | | | | 3 months later—controlled at 7000 | | Good | 98 |
| A.M. | M | 68 | 2 | Chlorambucil | 115000 | 13000 | Good | 101 |
| R.P. | M | 51 | 8 | Cyclo-phosphamide | *46000 | 2900 | Good | 94 |
| G.S. | M | 63 | 1 | Chlorambucil | 250000 | 10000 | Good | 100 |
| | | | | | *6 months later—controlled at 10000 | | Good | 97 |
| J.H. | M | 69 | 3 | Chlorambucil | 75000 | 6500 | Good | 88 |
| T.M. | F | 60 | 11 | Chlorambucil | 420000 | 3100 | Good | 99 |
| | | | | CLASS II | | | | |
| R.P. | M | 53 | 10 | Chlorambucil | 80000 | 80000 | Poor | 56 |
| L.R. | F | 67 | 11 | Cyclo-phosphamide | 100000 | 100000 | Poor | 65 |
| A.A. | M | 58 | 2 | Chlorambucil | 577000 | 385000 | Poor | 65 |
| | | | | | 6 months later 198000 | 114000 | Poor | 61 |
| I.F. | M | 69 | 25 | | No treatment given | | — | 51 |
| V.P. | F | 76 | 5 | | No treatment given | | — | 68 |
| P.S. | M | 60 | >7 | Chlorambucil and cyclo-phosphamide | 75000 | 60000 | Poor | 70 |
| G.S. | M | 66 | 4 | Chlorambucil | 3 years after initial sample* 45000 | 4000 | Good | 63 |
| T.H. | M | 60 | >6 | Cyclo-phosphamide | 4500 | 18000 | Poor | 54 |
| E.D. | F | 72 | >5 | Cyclo-phosphamide | 3900 | 6360 | Poor | 66 |

* See also CLASS II

treatment (Class I), little change occurred in the aromatic absorption of intracellular chlorambucil. On the other hand, when the aromatic structure of the drug was altered, in the *in vitro* assay, this could be correlated with an unsatisfactory clinical course or poor response to treatment (Class II).

It should be noted that lymphocytes from R.P., when first tested, failed to degrade chlorambucil and at this time the patient was responding to treatment. When examined one year later lymphocytes from this patient *did* degrade the drug and treatment then proved clinically ineffective. In the case of G.S., *in vitro* testing on 2 occasions in 1968 suggested that this patient would be sensitive to chlorambucil, and this proved to be the case clinically. However, subsequent tests 3 years later indicated that this patient's lymphocytes were now degrading the drug though G.S. continued to respond to treatment. Although this behaviour represents the single exception to the classification presented above, G.S. will be followed up on this *in vitro* screen in the event that he may subsequently develop resistance to treatment.

This simple test procedure may provide a rapid means of assessing a patient's potential response to chemotherapy. Recently other workers have proposed an *in vitro* assay system to detect the sensitivity of CLL lymphocytes to chlorambucil (Lawler, Lele and Pentycross, 1971). This procedure is based on the survival of cells cultured continuously in the presence of the drug. Its disadvantage is the greater time required (5 days) for assessment of response. The present method provides a result in a few hours.

The authors are indebted to Drs D. A. G. Galton and E. Wiltshaw for providing blood samples from their patients for this study. One of us (B. T. H.) acknowledges the receipt of a Wellcome Foundation Postdoctoral Fellowship. This investigation has been supported by grants to the Chester Beatty Research Institute (Institute of Cancer Research: Royal Cancer Hospital) from the Medical Research Council and the Cancer Research Campaign.

### REFERENCES

EPSTEIN, J., ROSENTHAL, R. A. & ESS, R. J. (1955) Use of $\gamma$-(4-Nitrobenzyl)pyridine as an Analytical Reagent for Ethylenimines and Alkylating Agents. *Analyt. Chem.*, **27**, 1435.

HARRAP, K. R. & HILL, B. T. (1970) The Selectivity of Action of Alkylating Agents and Drug Resistance. III. The Uptake and Degradation of Alkylating Drugs by Yoshida Ascites Sarcoma Cells *in vitro*. *Biochem. Pharmac.*, **19**, 209.

LAWLER, S. D., LELE, K. P. & PENTYCROSS, C. R. (1971) A Simple *in vitro* test for Assessing the Sensitivity of Lymphocytes to Chlorambucil. *Br. J. Cancer*, **25**, 493.